# Calcitriol Suppresses Warburg Effect and Cell Growth in Human Colorectal Cancer Cells

**DOI:** 10.3390/life11090963

**Published:** 2021-09-14

**Authors:** Chun-Yin Huang, Yu-Ting Weng, Po-Chen Li, Nien-Tsu Hsieh, Chun-I Li, Hsiao-Sheng Liu, Ming-Fen Lee

**Affiliations:** 1 Department of Nutrition, China Medical University, Taichung 406040, Taiwan; chuang@mail.cmu.edu.tw (C.-Y.H.); u106076201@cmu.edu.tw (Y.-T.W.); u107076003@cmu.edu.tw (P.-C.L.); u102059651@cmu.edu.tw (N.-T.H.); 2 Department of Nutrition and Health Sciences, Chang Jung Christian University, Tainan 711301, Taiwan; 3 Department of Microbiology and Immunology, National Cheng Kung University, Tainan 70101, Taiwan; cili@glyconex.com.tw (C.-I.L.); hsliu713@kmu.edu.tw (H.-S.L.); 4 Center for Cancer Research, Graduate Institute of Clinical Medicine, College of Medicine, Kaohsiung Medical University, Kaohsiung 80708, Taiwan

**Keywords:** Warburg effect, cell growth, calcitriol, vitamin D, colorectal cancer

## Abstract

Increasing lines of evidence indicate that the biologically active form of vitamin D, calcitriol (1,25-dihydroxyvitamin D_3_), prevents cancer progression by reducing cell proliferation, increasing cell differentiation, and inhibiting angiogenesis, among other potential roles. Cancer cells in solid tumors preferably undergo the “Warburg effect” to support cell growth by upregulating glycolysis, and the glycolytic intermediates further serve as building blocks to generate biomass. The objective of the current study is to investigate whether calcitriol affects glucose metabolism and cell growth in human colorectal cancer cells. Calcitriol reduced the expression of cyclin D1 and c-Myc. In addition, calcitriol reduced the expression of glucose transporter 1 (GLUT1) and key glycolytic enzymes and decreased extracellular acidification rate but increased oxygen consumption rate in human colorectal cancer cells. In a subcutaneous HT29 xenograft NOD/SCID mouse model, the volume and weight of the tumors were smaller in the calcitriol groups as compared with the control group, and the expression levels of GLUT1 and glycolytic enzymes, hexokinase 2 and lactate dehydrogenase A, were also lower in the calcitriol groups in a dose-responsive manner. Our data indicate that calcitriol suppresses glycolysis and cell growth in human colorectal cancer cells, suggesting an inhibitory role of the biologically active form of vitamin D in colorectal cancer progression.

## 1. Introduction

Vitamin D, an essential nutrient responsible for calcium homeostasis, also exhibits important extraskeletal, noncalcemic functions [1]. Calcitriol, or 1,25-dihydroxyvitamin D_3_ (1,25(OH)_2_D_3_), the biologically active form of vitamin D, exerts pleiotropic genomic and nongenomic actions [2]. Vitamin D often exerts its effect through the vitamin D receptor (VDR), a nuclear receptor superfamily member, by interacting with other transcription factors [3]. Upon entering the cells, calcitriol interacts with VDR, which heterodimerizes with retinoid X receptors (RXRs). The complex then translocates into the nucleus and binds to the vitamin D response element (VDRE) in the promoters of the target genes to regulate gene expression. Calcitriol transduces signals through related nuclear receptor members and regulates many common aspects of physiological function, including cell growth and differentiation in epithelial tissues and the immune system [2,4]. Increasing lines of evidence indicate potent anticancer activities in in vitro and in vivo animal models for 1,25(OH)_2_D_3_ and its analogs [2]. Calcitriol may prevent cancer progression by multiple levels, including reducing cell proliferation, increasing cell differentiation and apoptosis [1,5,6,7], and inhibiting angiogenesis through the hypoxia-inducible factor-1 (HIF-1) pathway [8].

According to GLOBOCAN 2018, for both sexes combined, colorectal cancer is the third most commonly diagnosed for cancer incidence and the second leading cause for cancer mortality [9]. Canonical Wnt/β-catenin signaling regulates a whole spectrum of physiological functions during embryogenesis and in adult tissues, and consequently, mutations of the main players and subsequent dysregulation of the Wnt signaling pathway can lead to cancer development. Specifically, constitutive activation of the Wnt/β-catenin pathway is one common theme during the progression of colorectal cancer (CRC). β-Catenin, one critical factor in the pathway, may reside in multiple subcellular locations, including adherens junction, cytoplasm, and nucleus in colonocytes [10]. Its role in adherens junction, together with E-cadherin and other adherens proteins, helps to stabilize the morphology of the epithelial cells. A β-catenin destruction complex, mainly containing adenomatous polyposis coli (APC), glycogen synthase kinase 3β (GSK3β), Axin, and casein kinase 1 (CK1), works in concert to regulate the cellular fate of β-catenin through sequential phosphorylation and proteasomal degradation, thereby controlling the activation of the Wnt/β-catenin signaling [11]. Once the activation signal is transmitted, β-catenin escapes from the disruption complex and moves into the nucleus. Nuclear β-catenin then enter into transactivation of the downstream target genes, including cyclin D1 and c-Myc [12], by binding to the transcription factors, T cell factor (TCF) and lymphoid enhancer factor (LEF) [13]. If the components of the destruction complex malfunction, they can no longer gatekeep the nuclear translocation of β-catenin, which may ultimately predispose to colorectal cancer [10].

Metabolic reprogramming is considered as one of the hallmarks of cancer, and the phenomenon of the “Warburg effect,” in which cancer cells employ aerobic glycolysis to supply energy and biosynthetic building blocks, has gained the most attention. Normal cells mainly rely on mitochondrial oxidative phosphorylation to generate energy, whereas cancer cells preferentially rely on the Warburg effect even in an oxygen-rich environment [14]. Although glycolysis generates much less energy than that of oxidative phosphorylation, cancer cells compensate for this disadvantage by accelerating the uptake of glucose and increasing the rate of glycolysis [15]. In addition, previous studies have indicated that several proteins, including glucose transporter 1 (GLUT1), hexokinase 2 (HK2), and lactate dehydrogenase A (LDHA), in glycolysis are often overexpressed in cancer [14,16]. The upregulation of these enzymes enhances the efficiency of glycolysis. The glycolytic intermediates are also providers for the synthesis of macromolecules, DNA, protein, and lipid to build up the biomass [17].

The current study aimed to investigate the effect of biologically active vitamin D on cell growth and the Warburg effect in colorectal cancer. We found that calcitriol inhibited cell growth and suppressed the expression of GLUT1 and glycolytic proteins in human colorectal cancer cells in vitro and in vivo, indicating a protective role of calcitriol in colorectal cancer.

## 2. Materials and Methods

### 2.1. Cell Culture

All cell culture supplies were purchased from Invitrogen, unless otherwise specified. HT29 and SW480, two human colorectal cancer cell lines, were obtained from the American Type Culture Collection (Manassas, VA, USA). HT29 cells were maintained in Dulbecco’s modified Eagle’s medium containing 10% fetal bovine serum (FBS, Gibco, Thermo Fisher Scientific, Inc., Waltham, MA, USA); SW480 cells were cultured in Leibovitz’s L-15 medium containing 10% FBS without CO_2_.

### 2.2. Chemicals, Reagents, and Antibodies

Most chemicals and reagents were purchased from Sigma (St. Louis, MO, USA), unless indicated otherwise. Calcitriol, the active form of vitamin D_3_ (1,25-dihydroxyvitamin D_3_), was obtained from Cayman Chemical (Ann Arbor, MI, USA) and dissolved in dimethyl sulfoxide (DMSO). Antibodies for cyclin D1, c-Myc, hexokinase 2 (HK2), lactate dehydrogenase A (LDHA), and β-actin were from GeneTex, Inc. (Irvine, CA, USA); anti-α-tubulin and anti-GAPDH were from Santa Cruz Biotechnology (Santa Cruz, CA, USA). Antibodies for glucose transporter 1 (GLUT1) and E-cadherin were from Cell Signaling Technology (Beverly, MA, USA) and Abcam (Cambridge, MA, USA), respectively. Antibodies for β-catenin as well as horseradish peroxidase (HRP)-conjugated anti-mouse and anti-rabbit secondary antibodies were obtained from EMD Millipore (Billerica, MA, USA). ECL detection reagents and polyvinylidene difluoride membranes were from Biokit Biotechnology Inc. (Miaoli, Taiwan) and Perkin Elmer Life Sciences, Inc. (Waltham, MA, USA), respectively. The Dual-light^®^ system was obtained from Applied Biosystems (Foster City, CA, USA). The RSV-β-galactosidase plasmid was kindly provided by Dr. Amy Yee (Tufts University, MA, USA).

### 2.3. Cell Viability Assay

Cell viability was determined by 3-(4,5-dimethylthiazole-2-yl)-2,5-biphenyltetrazolium bromide (MTT) assay, as described before [18]. Human colorectal cancer cells were seeded in 96-well culture plates and, the next day, incubated with calcitriol (0–1000 nM) for 48 h. The reaction was concluded by the addition of MTT reagent and incubated at 37 °C for 3 h. The experiment was completed by measuring the absorbance at 570 nm by an enzyme-linked immunosorbent assay (ELISA) reader (Dynatech Laboratories, Chantilly, VA, USA).

### 2.4. Cell Lysates and Western Blot Analysis

Cells were extracted by 1% Nonidet P-40 (NP-40) lysis buffer, which contained 1% NP-40, 150 mM NaCl, 50 mM Tris, and protease and phosphatase inhibitors. Fifty micrograms of cell lysates were separated by SDS−PAGE and transferred onto polyvinylidene difluoride. The membranes were subsequently incubated with specific antibodies overnight at 4 °C, followed by corresponding horseradish peroxidase-conjugated secondary antibodies. The signal was detected by ECL solution, and the images were analyzed by ChemiDocTM XRS+ system from (Bio-Rad, Hercules, CA, USA).

### 2.5. Immunofluorescence Analysis

Cells (5 × 10^5^) were treated with calcitriol or vehicle control for 48 h, fixed with 4% paraformaldehyde for 10 min, and then permeabilized with 0.25% Triton X-100 for 5 min. After reacting with anti-β-catenin antibodies for 2 h followed by secondary antibodies for 1 h in the dark, the slides were mounted with 4′,6-diamidino-2-phenylindole (DAPI) (Thermo Fisher Scientific, Waltham, MA, USA). The cells were then visualized with a confocal microscope (Leica Microsystems, Wetzlar, Germany).

### 2.6. TOPFlash/FOPFlash Luciferase Reporter Assay

Cells were plated at a density of 5 × 10^4^ cells/well on 24-well culture plates, and calcitriol or vehicle control was added after 12 h of plating. After 24 h, cells were co-transfected with 0.5 μg of plasmid, containing 0.4 μg of either TOPFlash or FOPFlash luciferase reporter construct and 0.1 μg of RSV-β-galactosidase plasmid. Cell lysates were collected in lysis solution, and the activities of luciferase and β-galactosidase were measured by a Dual-Light^®^ System (Applied Biosystems, Bedford, MA, USA). Relative luciferase activity indicated the ratio of TOPFlash/FOPFlash luciferase activity relative to the corresponding β-galactosidase activity as compared with vehicle only.

### 2.7. Measurements of Cellular Respiration and Glycolysis

The Seahorse XFe24 Analyzer was employed to measure the status of cellular respiration and glycolysis by the Cell Mito Stress Test Kit and Glycolysis Kit, respectively, according to the manufacturer’s instructions (Agilent Technologies, Inc., Santa Clara, CA, USA). Briefly, HT-29 and SW480 cells were subjected to the addition of calcitriol or DMSO and seeded onto 24-well plates. Glucose (10 mM), oligomycin (1 µM), and 2-deoxy-D-glucose (2-DG, 75 mM) were added to measure the extracellular acidification rate (ECAR), and oligomycin (1 µM), carbonyl cyanide 4-(trifluoromethoxy)phenylhydrazone (FCCP) (0.5 µM), and rotenone/antimycin A (0.5 µM) were treated sequentially to measure oxygen consumption rate (OCR). Data were normalized to the respective cellular protein concentration and analyzed by Seahorse Wave Software. The relative levels of glycolysis and glycolytic capacity were calculated based on the ECAR data obtained from the Glycolysis test, whereas the relative levels of basal respiration, ATP production, maximal respiration, and spare capacity were calculated based on the OCR data obtained in the Cell Mito Stress test.

### 2.8. Xenograft Mice

Three-week-old male NOD/SCID mice were purchased from National Cheng Kung University Animal Center (Tainan, Taiwan). The animal study protocol (CJCU-104-001) was reviewed and approved by the Institutional Animal Care and Use Committee (IACUC) at Chang Jung Christian University, and all procedures performed in the animal study were in accordance with the ethical standards of the institution. The animals were given free access to autoclaved food and water during the study. After one week of acclimation, animals were implanted subcutaneously with HT29 cells (5 × 10^6^) to the flank regions and randomly assigned into the control or calcitriol (0.5 µg or 1 µg) groups with four mice in each group. Two weeks after the implantation, the animals were intraperitoneally administered calcitriol (0.5 µg or 1 µg per injection) or vehicle only every other day. Tumor size was recorded every other day for 4 weeks until sacrifice. Tumor volume = ½(length × width^2^). The expression levels of GLUT1, HK2, and LDHA were analyzed by Western blotting.

### 2.9. Statistical Analysis

Data are presented as means ± standard errors of the mean (SEM), which were analyzed by Student’s *t*-test or the analysis of variance (ANOVA) test followed by Tukey’s post hoc comparison to analyze the differences between groups as appropriate. The results were considered significantly different at *p* < 0.05.

## 3. Results

### 3.1. Effect of Calcitriol on Cell Viability and the Expression of Cell Growth-Associated Proteins in Human Colorectal Cancer Cells

**Figure 1:**
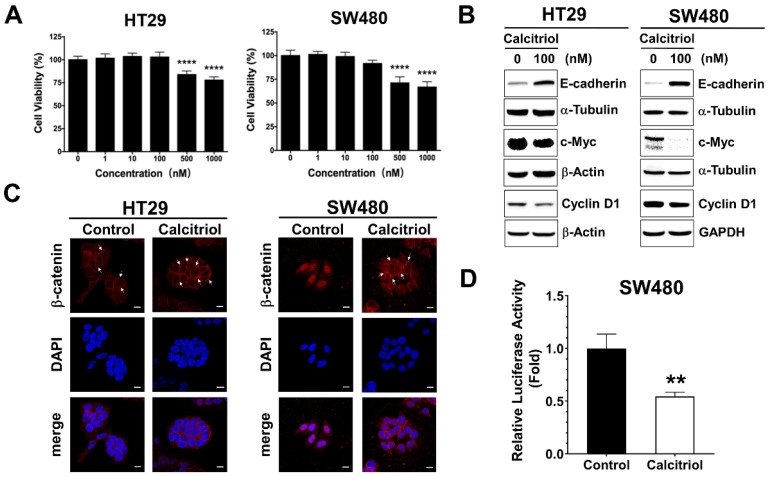
Calcitriol affected the expression of E-cadherin and Wnt/β-catenin target genes in human colorectal cancer cells. (**A**): HT29 and SW480 cells were treated with various doses of calcitriol for 48 h and subjected to MTT assay. Data are expressed as means ± standard error of the mean (SEM), *n* = 3; **** *p* < 0.0001 as compared with the vehicle control. B-D: Cells were treated without or with calcitriol (100 nM) for 48 h and subjected to various assays. (**B**): Western blotting (the original, unprocessed data included in Supplementary Materials Figure S1). (**C**): Immunofluorescence assay. After incubating with anti-beta-catenin antibodies and secondary antibodies, the slides were then mounted with DAPI-containing mounting medium. Representative images were taken under 630× magnification. Scale bar = 10 µm. (**D**): Luciferase reporter assay. Cells were co-transfected with TOPFlash or FOPFlash luciferase reporter genes and RSV-β-galactosidase construct for 24 h. Relative luciferase activity was the ratio of TOPFlash/FOPFlash luciferase activity after normalization with β-galactosidase activity, ** *p* < 0.01 as compared with the vehicle control.

The cell viability of the human colorectal cancer cells upon calcitriol treatment was evaluated by the MTT assay. There was no statistical difference in cell viability up to 100 nM, whereas 500 nM and above of calcitriol treatment resulted in cell death in both HT29 and SW480 human colorectal cancer cells, as shown in Figure 1A. Colorectal cancer cells often exhibit active Wnt/β-catenin signaling, and previous literature reported that calcitriol at a concentration of 100 nM for 48 h appears to upregulate the expression of E-cadherin and modulate β-catenin signaling in SW480 cells [19]. Therefore, without affecting cell viability, also to eliminate the potential complexity of cell death such as apoptosis or autophagy impinging on cells, we chose the condition of 100 nM of calcitriol for 48 h for the subsequent experiments. In addition to SW480, as reported previously [19], calcitriol increased the expression of E-cadherin in HT29 cells (Figure 1B). Calcitriol decreased the expression of cyclin D1 and c-Myc, two downstream target genes of the Wnt/β-catenin signaling in both HT29 and SW480 cells (Figure 1B). Furthermore, calcitriol caused the majority of β-catenin translocated from the nucleus to the cell membrane in SW480 cells (Figure 1C); calcitriol also significantly reduced the activation of the Wnt/β-catenin signaling in SW480 cells (Figure 1D), as shown by the immunofluorescence approach and the TOPFlash/FOPFlash reporter system. However, albeit a tendency of enhanced membrane localization of β-catenin upon calcitriol addition, we did not observe prominent nucleus-to-membrane translocation of β-catenin in HT29 cells (Figure 1C); neither did we observe a significant reduction of the TOPFlash/FOPFlash reporter system as that of SW480 cells. These data indicated that calcitriol may suppress cell growth partially through the Wnt/β-catenin signaling in human colorectal cancer cells.

### 3.2. Effect of Calcitriol on Glycolysis and Mitochondrial Respiration in Human Colorectal Cancer Cells

**Figure 2:**
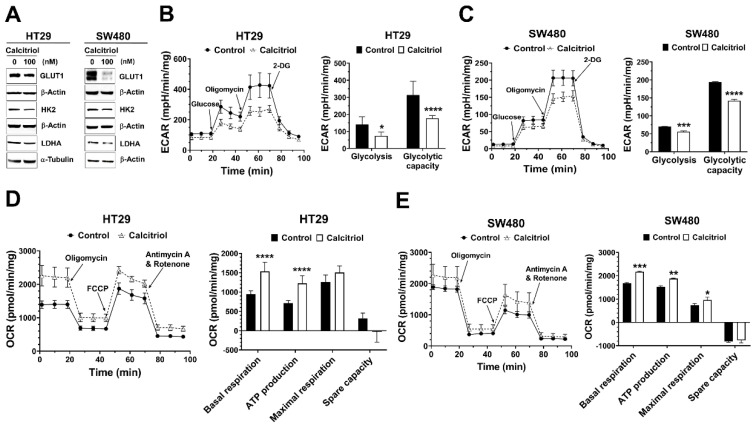
Calcitriol affected the expression of glycolysis-related proteins, extracellular acidification rate, and oxygen consumption rate in human colorectal cancer cells. HT29 and SW480 cells were treated without or with calcitriol (100 nM) for 48 h and subjected to various assays. (**A**): Western blotting (the original, unprocessed data included in Supplementary Materials Figure S2). (**B**,**C**): Analysis of extracellular acidification rate (ECAR) by Seahorse XF24 analyzer. Representative images of ECAR. Glucose, oligomycin, and 2-deoxyglucose (2-DG) were added at the indicated points (left panel). The quantification of glycolysis and glycolytic capacity of the ECAR were calculated as shown (right panel). (**D**,**E**): Analysis of oxygen consumption rate (OCR) by Seahorse XF24 analyzer. Representative images of OCR where oligomycin, carbonyl cyanide 4-(trifluoromethoxy)phenylhydrazone (FCCP), and rotenone/antimycin A were sequentially added as indicated (left panel). The quantification of basal and maximal respiration, ATP production, and spare capacity of OCR were calculated as shown (right panel). Data are expressed as means ± standard error of the mean (SEM), *n* = 3; * *p* < 0.05, ** *p* < 0.01, *** *p* < 0.001, **** *p* < 0.0001 as compared with the vehicle control.

Recent studies have revealed that several oncogenic signaling pathways regulate cancer metabolism by controlling the expression and/or activity of metabolic enzymes. Aerobic glycolysis is one major feature in solid tumors, and several glycolysis-related proteins are often dysregulated in cancer [14,16]. Therefore, we assessed the effect of calcitriol on the expression of glucose transporter 1 (GLUT1) and several glycolytic proteins. Calcitriol suppressed the protein expression levels of GLUT1 in both SW480 and HT29 cells (Figure 2A). Calcitriol reduced the expression of hexokinase 2 (HK2) and lactate dehydrogenase A (LDHA) in HT29 cells but not as prominently in SW480 cells (Figure 2A). Further seahorse cellular bioenergetics analysis demonstrated that calcitriol decreased extracellular acidification rate (ECAR) in terms of glycolysis and glycolytic capacity (Figure 2B,C) but increased oxygen consumption rate (OCR) in the aspects of basal respiration, ATP production, and maximal respiration (Figure 2D,E), in both HT29 and SW480 cells, indicating that these cells switched their metabolic mode from aerobic glycolysis towards more mitochondrial respiration in the presence of calcitriol. Together, calcitriol downregulated the Warburg effect and increased mitochondrial oxygen consumption in human colorectal cancer cells.

### 3.3. In vivo Effect of Calcitriol on Colorectal Tumor Growth and Glycolysis

**Figure 3:**
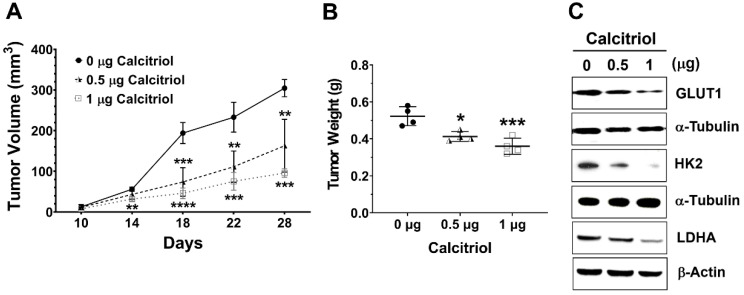
Calcitriol reduced tumor volume and tumor weight and decreased the expression of the glycolytic proteins of the subcutaneous HT29 xenografts. Four-week-old male NOD/SCID mice were implanted subcutaneously with HT29 cells (5 × 10^6^) to the flank regions and randomly assigned into the control or calcitriol (0.5 µg or 1 µg) groups (*n* = 4 in each group). Two weeks after the implantation, the animals were administered vehicle only (0 µg) or calcitriol (0.5 µg or 1 µg) by intraperitoneal injection with the respective dose, 0 µg, 0.5 µg, or 1 µg of calcitriol for each animal every other day. Tumor size was recorded every other day for 4 weeks until sacrifice. (**A**,**B**): Tumor volume and tumor weight of the xenografts. Data are expressed as means ± standard error of the mean (SEM); * *p* < 0.05, ** *p* < 0.01, *** *p* < 0.001, **** *p* < 0.0001 as compared with the vehicle control (0 µg). (**C**): The expression levels of GLUT1, HK2, and LDHA in the xenografts were analyzed by Western blotting (the original, unprocessed data included in Supplementary Materials Figure S3).

Next, we employed a subcutaneous HT29 xenograft NOD/SCID mouse model to investigate the in vivo effect of calcitriol on cell growth and glycolysis. Tumor volume and weight in the control group were significantly higher than those in the calcitriol groups, and we observed a dose-dependent trend of calcitriol in reducing tumor volume and weight (Figure 3A,B). Moreover, calcitriol dose-dependently suppressed the expression of glycolysis-associated proteins, including GLUT1, HK2, and LDHA, in the subcutaneous xenografts (Figure 3C). The in vivo findings were consistent with the in vitro data that calcitriol suppressed glycolysis and cell growth in human colorectal cancer cells.

## 4. Discussion

In the current study, we found that calcitriol reduced the expression of cyclin D1 and c-Myc. Calcitriol also reduced the expression of GLUT1 and key glycolytic enzymes, decreased extracellular acidification rate, and increased oxygen consumption rate in human colorectal cancer cells. In addition, calcitriol reduced the tumor volume and weight of subcutaneous HT29 xenografts, accompanied by the decreased expression of glycolytic proteins. Our data indicate that calcitriol suppresses the Warburg effect and inhibits cell growth in human colorectal cancer cells.

It is generally considered that calcitriol tends to suppress cell growth and/or induce differentiation, among other roles, in human colorectal cancer cells [20,21]. We employed HT29 and SW480, two human colorectal cancer cell lines, to study the potentially beneficial effect of calcitriol, and we employed 100 nM of calcitriol for 48 h for the majority of the experiments. Evans et al. reported a significant dose-dependent, growth-inhibitory effect of calcitriol (10^−11^ to 10^−6^ M) in HT29 cells during a six-day period [22]. Pálmer et al. found that 100 nM of calcitriol for 48 h appears to promote differentiation in SW480 cells but not in SW620 cells [19]. Together, the effect of calcitriol on colorectal cancer cells may depend on cell type, the concentration of calcitriol, and its duration of treatment. Thomas et al. obtained rectal biopsy specimens from individuals with normal rectal mucosa and patients with familial adenomatous polyposis, and they established explants in organ culture to assess the effect of calcitriol on rectal epithelial proliferation by determining the crypt cell production rate and Ki-67 labeling index [23]. In both normal and premalignant human rectal specimens, calcitriol reduced crypt cell production rate and Ki-67 labeling index [23]. Therefore, calcitriol may also be able to suppress the proliferation of crypt cells.

Normally, β-catenin can be found mostly in the cell membrane, together with other cell–cell junction proteins, such as the cadherin family members, to maintain the integrity of the epithelium. Although the subcellular location of β-catenin between cell–cell junction and nucleus is not necessarily always coupled, it is possible that there is a crosstalk present between cadherin-mediated cell adhesion and Wnt/β-catenin signaling [24]. The downstream target genes of the Wnt/β-catenin signaling pathway, including cyclin D1 and c-Myc, are involved in cell cycle progression. In the current study, we employed two human colorectal cancer cell lines, SW480 and HT29. Both cell lines carry wild-type *CTNNB1* (β-catenin) and *CDH1* (E-cadherin), but mutant *APC* [25,26], and these cells exhibit active Wnt/β-catenin signaling. We found that calcitriol suppressed the expression of cyclin D1 and c-Myc, two downstream targets of the Wnt/β-catenin signaling pathway (Figure 1B). Interestingly, calcitriol reduced nuclear β-catenin and β-catenin-mediated transcriptional activity in SW480 cells, but this was not apparent in HT29 cells (Figure 1C,D). It has been shown that 1,25(OH)_2_D_3_ promotes the differentiation of colon cancer cells by stimulating E-cadherin and inhibiting β-catenin signaling [19]. Vitamin D appears to modulate the Wnt/β-catenin signaling in colon cancer whose dysregulation leads to the overexpression of cell division/metastatic genes and contributes to a highly proliferative, undifferentiated cell phenotype [27]. Calcitriol increased the expression of E-cadherin in both cell lines, with a more prominent increase in SW480 cells (Figure 1B). Larriba et al. reported that calcitriol had no additional effect on either the translocation or the transcriptional activity of β-catenin in HT29 cells probably due to higher endogenous expression of E-cadherin as compared with SW480 [28].

Several glycolysis-related proteins, for example, GLUT1, HK2, and LDHA, are often upregulated in several types of cancer, which are related to the Warburg effect and cancer progression [14,16]. Efficient glucose transporters can meet the high demands of glucose uptake to enhance the Warburg effect. Among glucose transporters, increased expression of GLUT1 is identified in multiple types of cancer, indicating higher glucose uptake in cancer [29,30]. Hexokinase (HK) catalyzes the first rate-limiting step of glycolysis, which phosphorylates glucose to generate glucose-6-phosphate (G-6-P). There are four HK isoforms in mammalian tissues, HK1, HK2, HK3, and HK4. HK2 overexpression can be found in cancer cells [31]. Because HK2 is not inhibited by G-6-P, this feature provides a competitive edge for glycolytic flux. LDH enzyme is a homotetramer or heterotetramer assembled by two subunits, M and H, which are encoded by LDHA and LDHB, respectively. LDH catalyzes the conversion of pyruvate to lactate. Several lines of evidence indicate that LDHA overexpresses in multiple types of cancer, including breast, colorectal, and non-small cell lung cancers [32]. In this study, we found that calcitriol decreased the expression of GLUT1 in vitro and in vivo (Figure 2A and Figure 3C), and both HT29 and SW480 colorectal cancer cells appeared to shun away from the Warburg effect by decreasing the extracellular acidification rate but increasing the oxygen consumption rate (Figure 3). A similar effect of calcitriol has been found in LNCaP prostate cancer cells such that calcitriol transcriptionally downregulates various glucose metabolism-related genes, including *SCL2A1* (GLUT1) and *LDHA*, and calcitriol also decreases glucose uptake and the expression of GLUT1 [33]. Therefore, among other roles, calcitriol may also hinder cancer progression by downregulating the proteins responsible for glycolytic flux.

Many studies have linked activated oncogenes and dysregulated transcription factors to altered glucose metabolism [34]. For example, *KRAS* mutation, *MYC* overexpression, and HIF1 transcription factor target several glycolytic genes, including *HK2* and *LDHA*. HT29 cells carry wild-type *KRAS* and SW480 cells carry *KRAS^G12V^*. In the current study, we found that calcitriol reduced the expression of GLUT1 in both SW480 and HT29 cells (Figure 2A); therefore, it appears that the inhibitory effect of calcitriol on GLUT1 was irrespective of *KRAS* mutation status. Recent studies have suggested crosstalk between the Wnt/β-catenin and c-Myc pathways in cancer cells such that these pathways unite to regulate cell cycle progression and metabolic reprogramming [35]. GLUT1 is a transactivating target gene of c-Myc [36]. LDHA, responsible for lactate production, is also a target gene of c-Myc and whose expression is necessary for c-Myc-mediated transformation [37]. Previous literature has indicated that β-catenin-mediated c-Myc expression upregulates several glycolytic genes, including *SLC2A1* (GLUT1) and *LDHA*, thereby promoting the Warburg effect in cancer cells [35]. Therefore, the suppressive effect of calcitriol on glycolytic proteins may be partially through inhibiting the β-catenin/c-Myc signaling.

## 5. Conclusions

**Figure 4:**
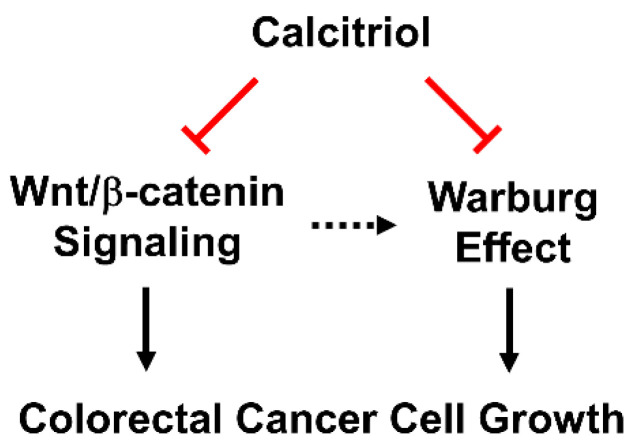
Calcitriol suppresses Wnt/β-catenin signaling and the Warburg effect, and, together, inhibits cell growth in human colorectal cancer cells.

We employed HT29 and SW480 to study the putatively beneficial roles of calcitriol, and we further investigated its in vivo effect by a subcutaneous xenograft model. The treatment of 100 nM of calcitriol for 48 h inhibited the β-catenin signaling by reducing the expression of c-Myc and cyclin D1 and suppressing the Warburg effect by decreasing the expression of several key glycolytic proteins in human colorectal cancer cells despite different genetic backgrounds. Consequently, calcitriol inhibited colorectal cancer cell growth (Figure 4). In addition, we confirmed the glycolysis-suppressive mode and the growth-inhibitory effect of calcitriol in the in vivo animal study. Therefore, vitamin D is not only an essential nutrient with critical physiological functions in a classical sense, but its biologically active form, calcitriol, also exhibits potential chemopreventive capacity.

## Data Availability

The data that support the findings of this study are available from the corresponding author upon reasonable request.

## Supplementary Materials

The following are available online at https://www.mdpi.com/article/10.3390/life11090963/s1, Figure S1. The original western blotting figures for Figure 1B Western blotting (A) HT29: E-cadherin, α-Tubulin. (B) HT29: c-Myc, β-Actin. (C) HT29: Cyclin D1, β-Actin. (D) SW480: E-cadherin, α-Tubulin. (E) SW480: c-Myc, β-Actin. (F) SW480: Cyclin D1, GAPDH. Figure S2. The original western blotting figures for Figure 2A Western blotting (A) HT29: GLUT1, β-Actin. (B) HT29: HK2, β-Actin. (C) HT29: LDHA, α-Tubulin. (D) SW480: GLUT1, β-Actin. (E) SW480: HK2, β-Actin. (F) SW480: LDHA, β-Actin. Figure S3. The original western blotting figures for Figure 3C Western blotting HT29 xenograft: (A) GLUT1, α-Tubulin. (B) HK2, α-Tubulin. (C) LDHA, β-Actin.
