# Supplementary material for: Calcitriol Suppresses Warburg Effect and Cell Growth in Human Colorectal Cancer Cells
Source: Life (Basel). 2021 Sep 14;11(9):963. doi: 10.3390/life11090963 (PMC8466965; doi:10.3390/life11090963)
Supplement: Supplementary file 1 [file life-11-00963-s001.zip › life-1315028-supplementary.pdf]

life

MDPI

Article

Calcitriol Suppresses Warburg Effect and Cell Growth in Human Colorectal Cancer Cells

Chun-Yin Huang [1], Yu-Ting Weng [1,2,†], Po-Chen Li [1,†], Nien-Tsu Hsieh [1], Chun-I Li [3], Hsiao-Sheng Liu [3,4] and Ming-Fen Lee [1,2,*]

[1] Department of Nutrition, China Medical University, Taichung 406040, Taiwan; chuang@mail.cmu.edu.tw (C.-Y.H.); u106076201@cmu.edu.tw (Y.-T.W.); u107076003@cmu.edu.tw (P.-C.L.); u102059651@cmu.edu.tw (N.-T.H.); leemf@cmu.edu.tw (M.-F.L.)
[2] Department of Nutrition and Health Sciences, Chang Jung Christian University, Tainan 711301, Taiwan
[3] Department of Microbiology and Immunology, National Cheng Kung University, Tainan 70101, Taiwan; cili@glyconex.com.tw (C.-I.L.); hsliu713@kmu.edu.tw (H.-S.L.)
[4] Center for Cancer Research, Graduate Institute of Clinical Medicine, College of Medicine, Kaohsiung Medical University, Kaohsiung 80708, Taiwan;
* Correspondence: leemf@cmu.edu.tw; Tel.: +886-4-2205-3366 (ext. 7510)
† These authors contributed equally.

Supplementary Material

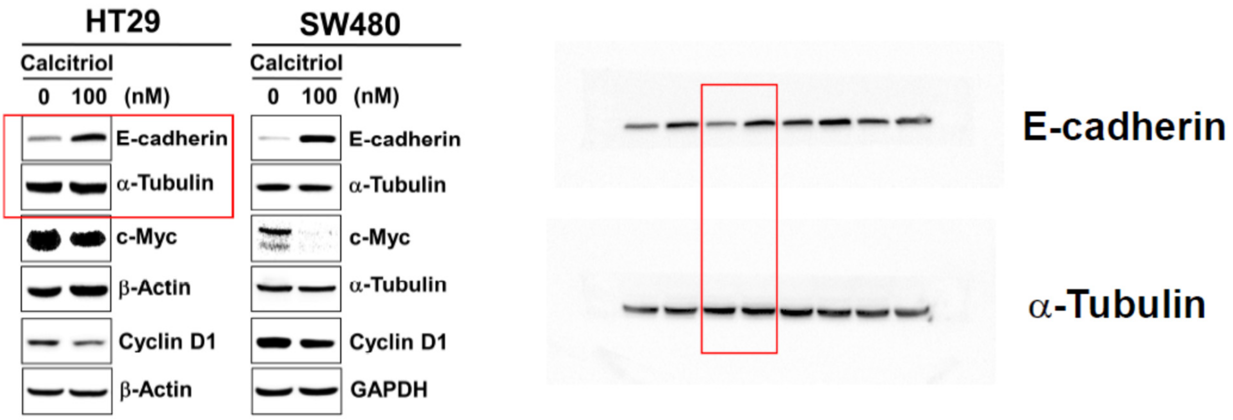

(A)

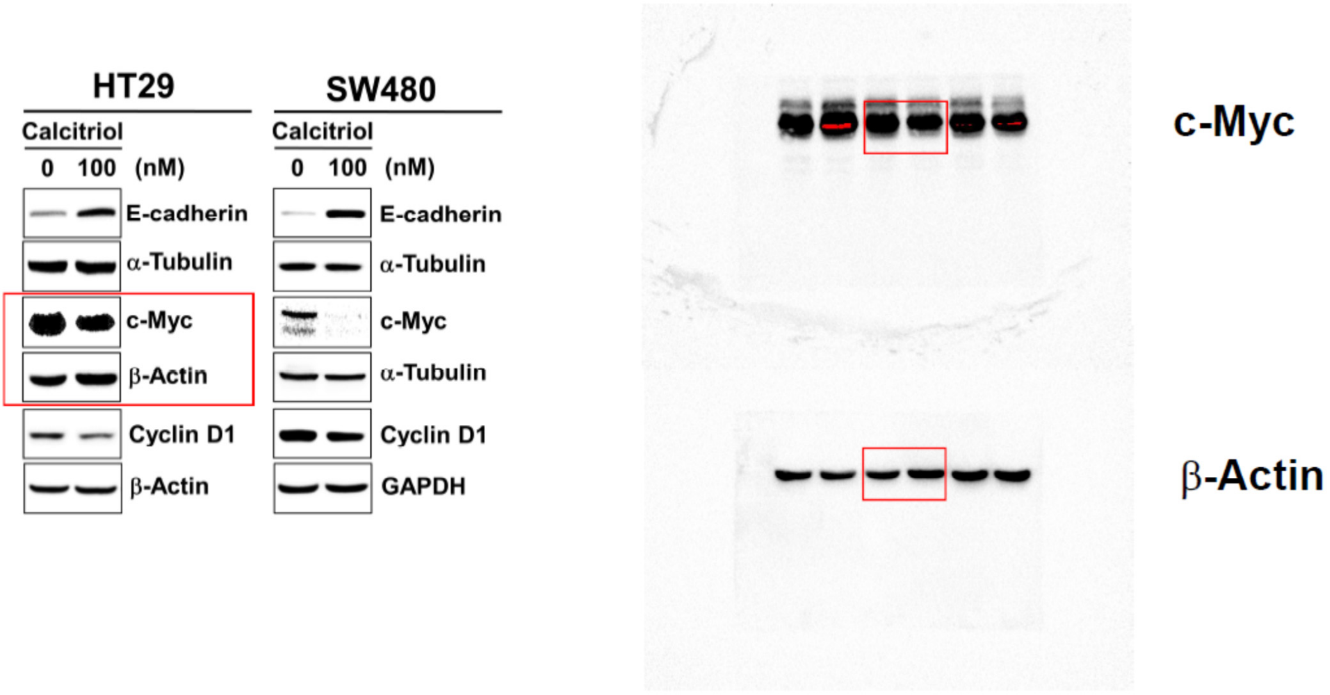

(B)

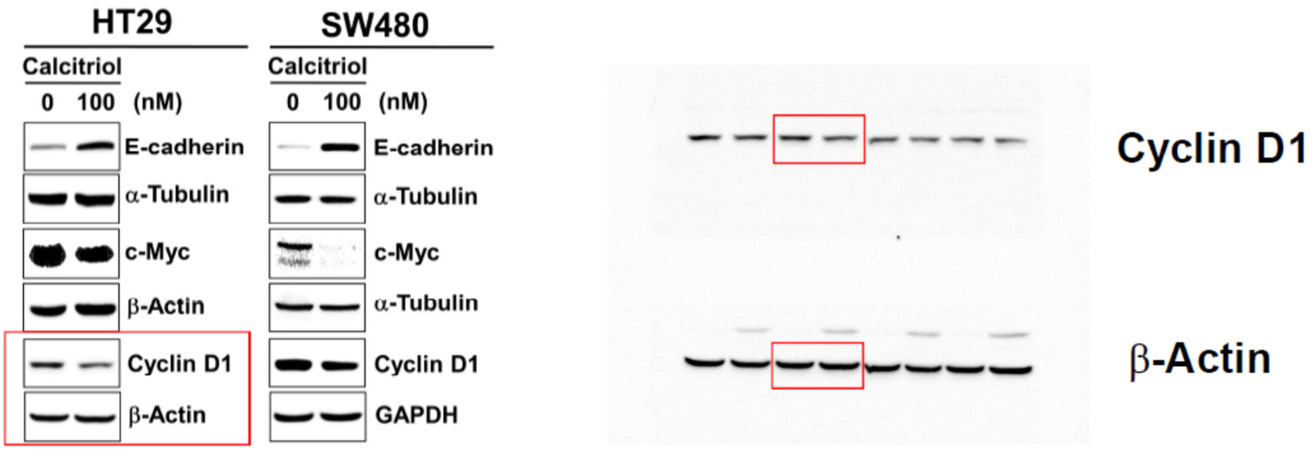
HT29
Calcitriol
0 100 (nM)
E-cadherin
α-Tubulin
c-Myc
β-Actin
Cyclin D1
β-Actin
SW480
Calcitriol
0 100 (nM)
E-cadherin
α-Tubulin
c-Myc
α-Tubulin
Cyclin D1
GAPDH
Cyclin D1
β-Actin

(C)

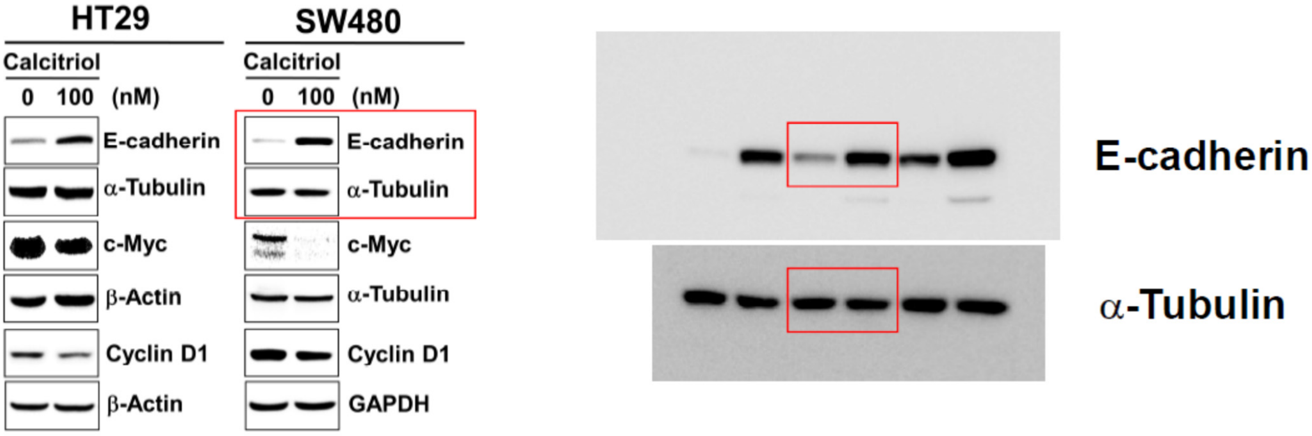
HT29
Calcitriol
0 100 (nM)
E-cadherin
α-Tubulin
c-Myc
β-Actin
Cyclin D1
β-Actin
SW480
Calcitriol
0 100 (nM)
E-cadherin
α-Tubulin
c-Myc
α-Tubulin
Cyclin D1
GAPDH
E-cadherin
α-Tubulin

(D)

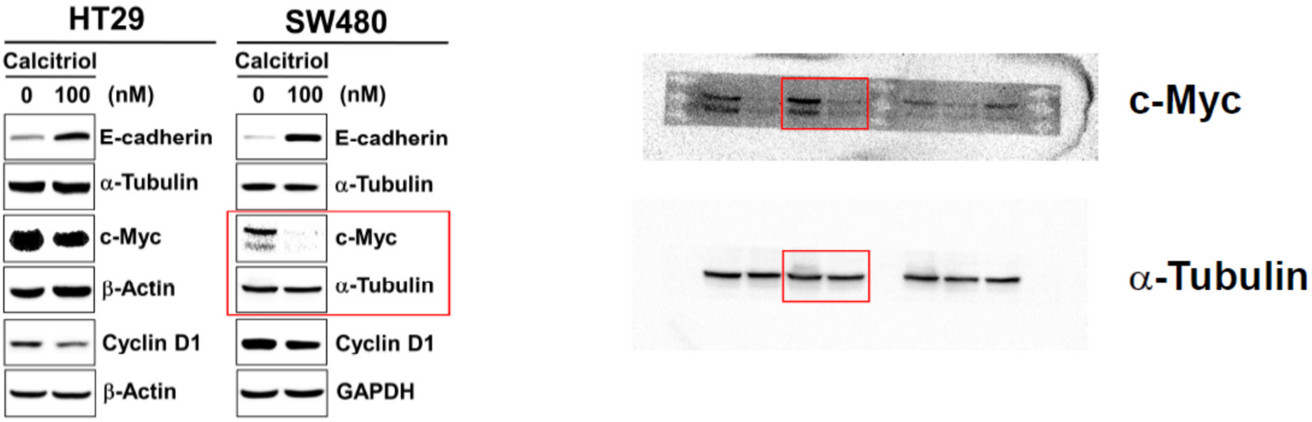
HT29
Calcitriol
0 100 (nM)
E-cadherin
α-Tubulin
c-Myc
β-Actin
Cyclin D1
β-Actin
SW480
Calcitriol
0 100 (nM)
E-cadherin
α-Tubulin
c-Myc
α-Tubulin
Cyclin D1
GAPDH
c-Myc
α-Tubulin

(E)

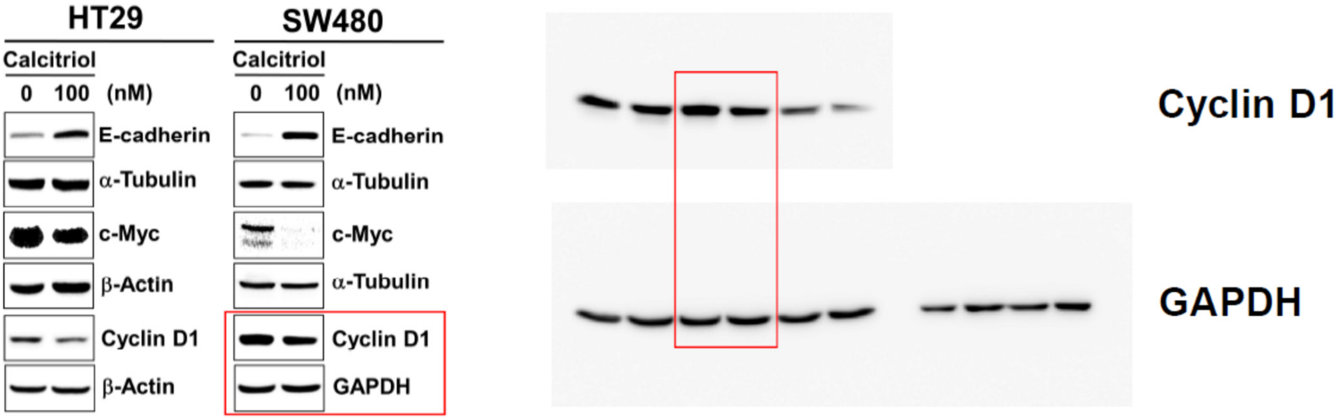

(F)

Figure S1. The original western blotting figures for Figure 1B Western blotting (**A**) HT29: E-cadherin, α-Tubulin. (**B**) HT29: c-Myc, β-Actin. (**C**) HT29: Cyclin D1, β-Actin. (**D**) SW480: E-cadherin, α-Tubulin. (**E**) SW480: c-Myc, β-Actin. (**F**) SW480: Cyclin D1, GAPDH.

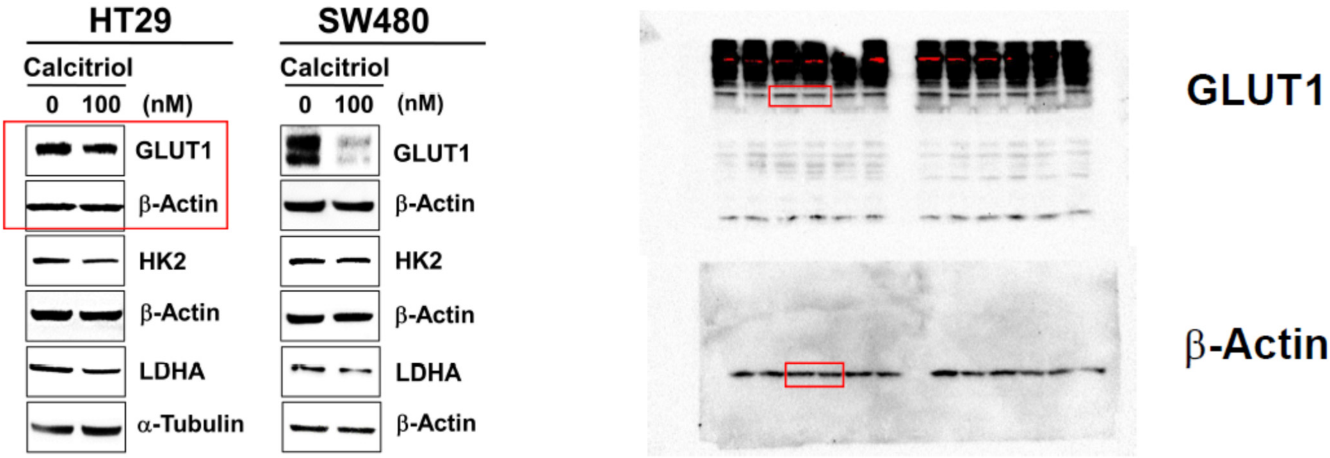

(A)

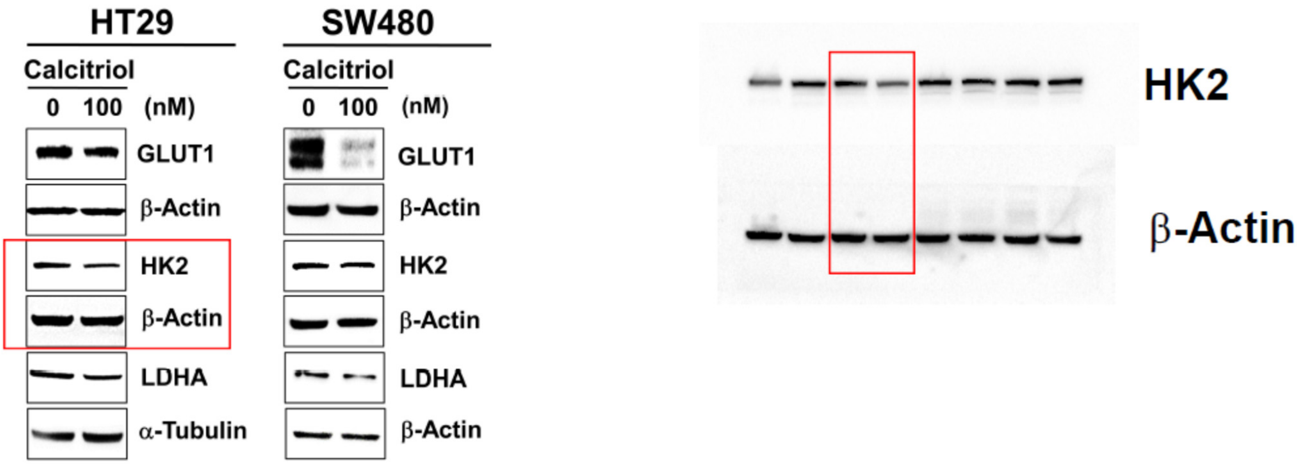

(B)

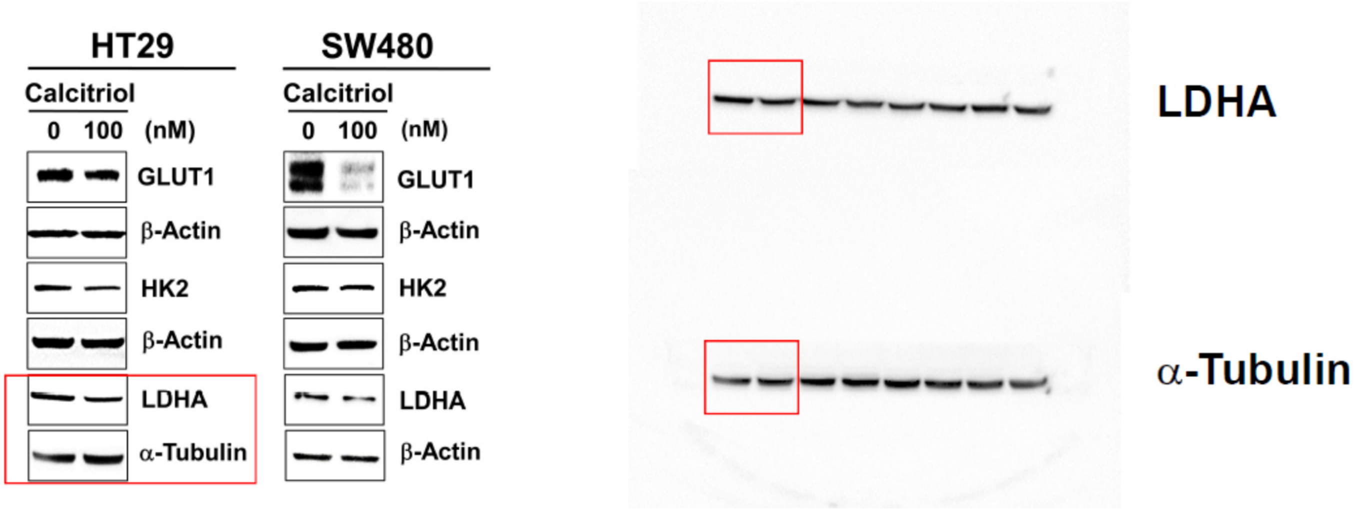
HT29
SW480
Calcitriol
0 100 (nM)
GLUT1
β-Actin
HK2
β-Actin
LDHA
α-Tubulin
LDHA
α-Tubulin

(C)

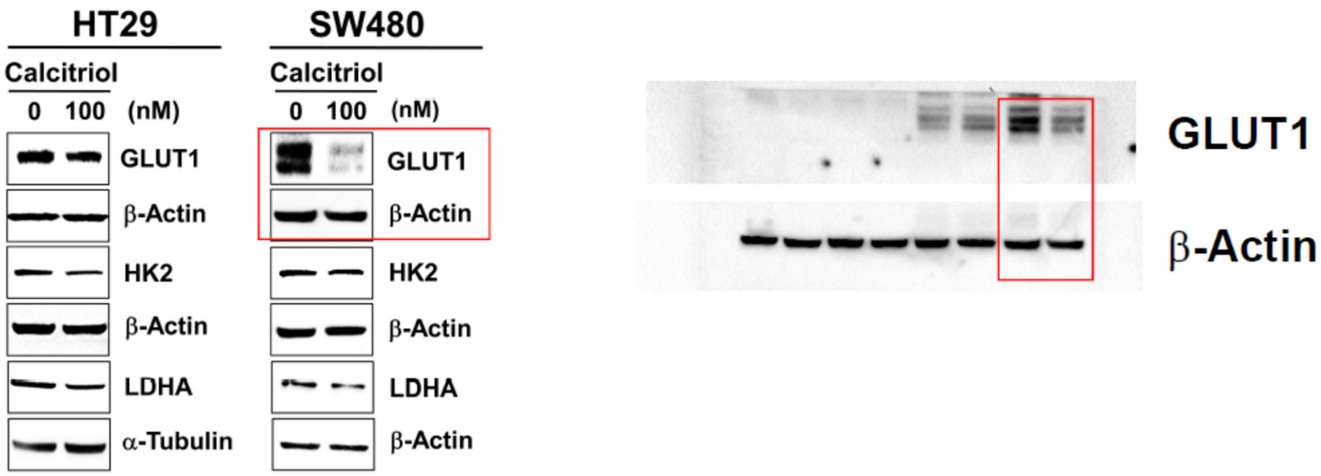
HT29
SW480
Calcitriol
0 100 (nM)
GLUT1
β-Actin
HK2
β-Actin
LDHA
α-Tubulin
GLUT1
β-Actin

(D)

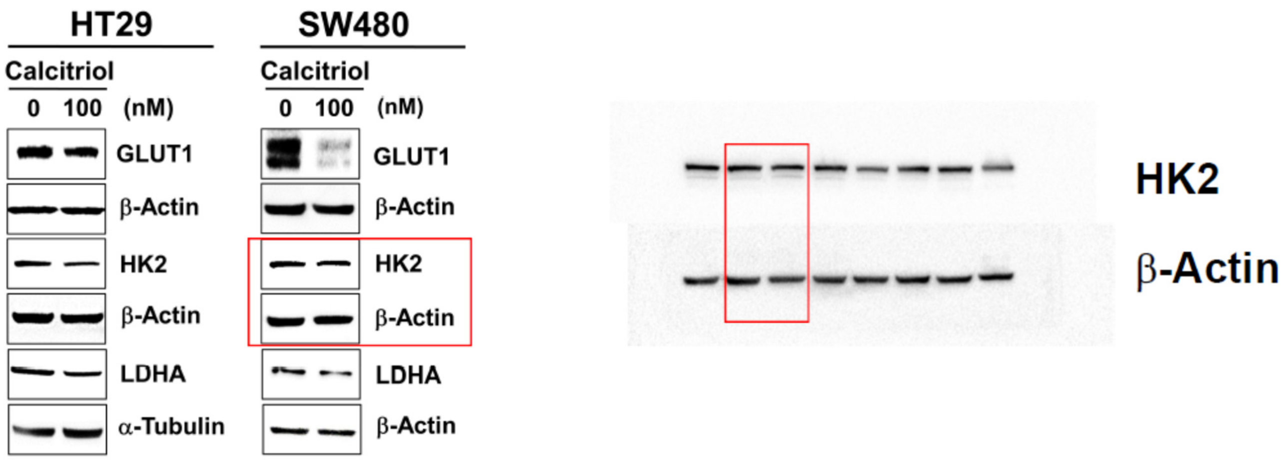
HT29
SW480
Calcitriol
0 100 (nM)
GLUT1
β-Actin
HK2
β-Actin
LDHA
α-Tubulin
HK2
β-Actin

(E)

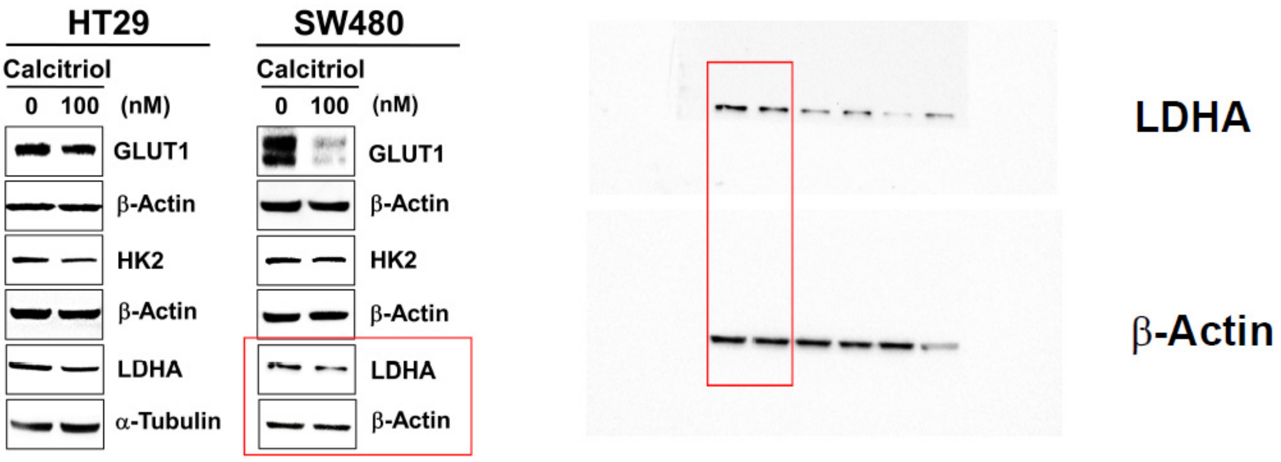

(F)

Figure S2. The original western blotting figures for Figure 2A Western blotting (**A**) HT29: GLUT1, β-Actin. (**B**) HT29: HK2, β-Actin. (**C**) HT29: LDHA, α-Tubulin. (**D**) SW480: GLUT1, β-Actin. (**E**) SW480: HK2, β-Actin. (**F**) SW480: LDHA, β-Actin.

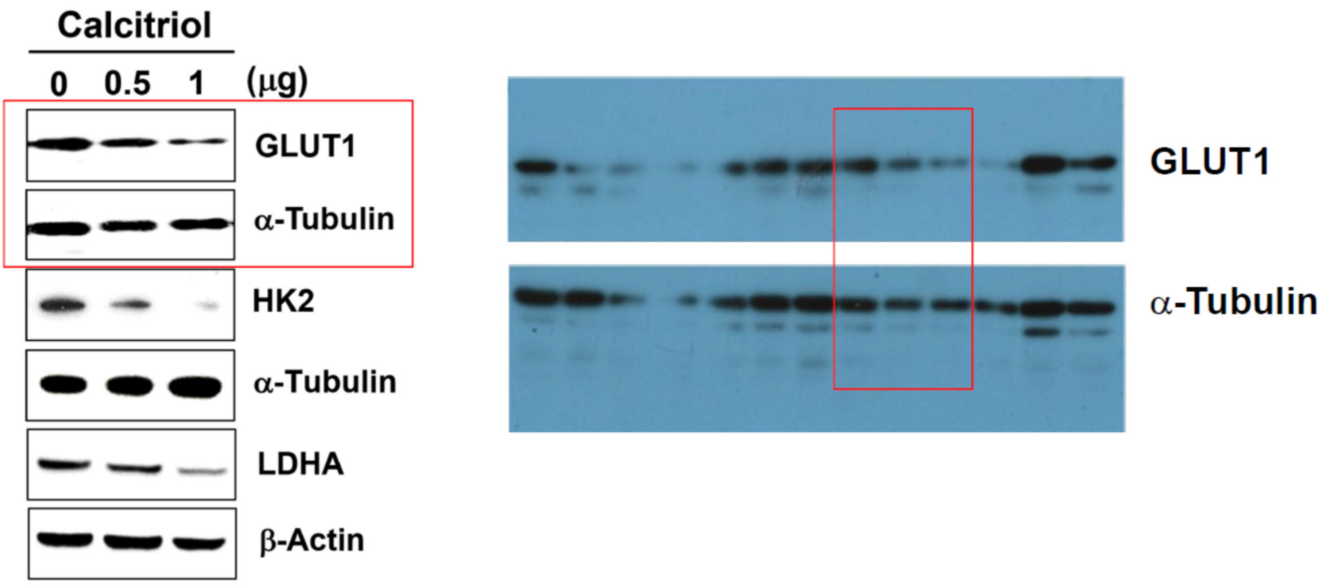

(A)

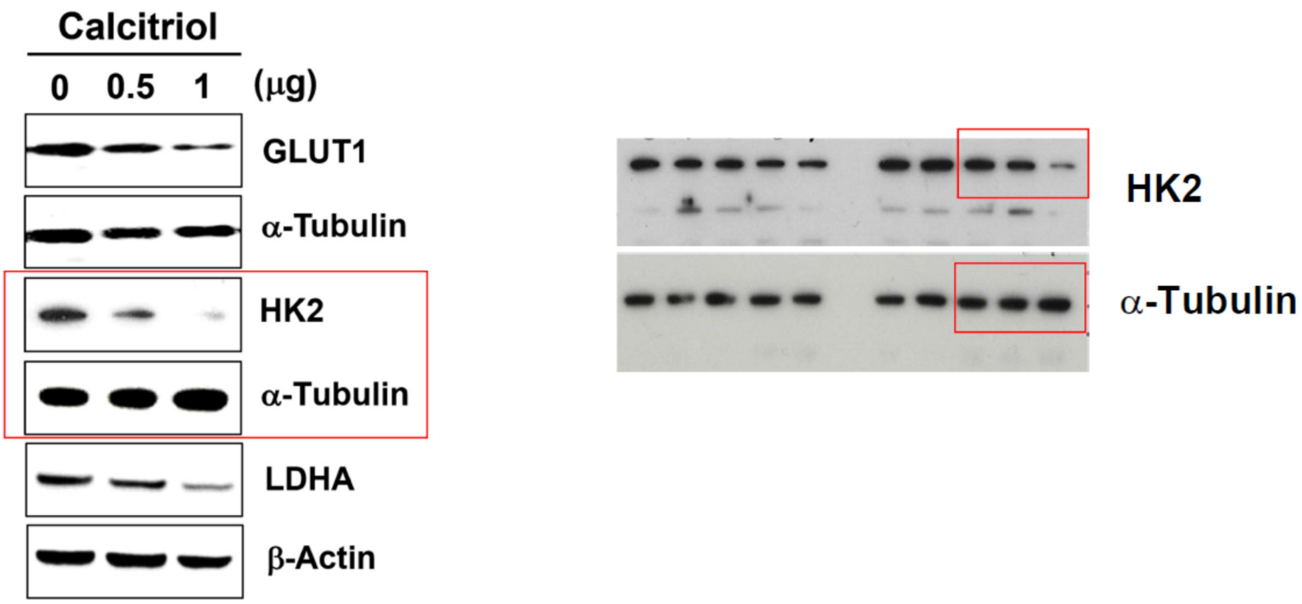

(B)

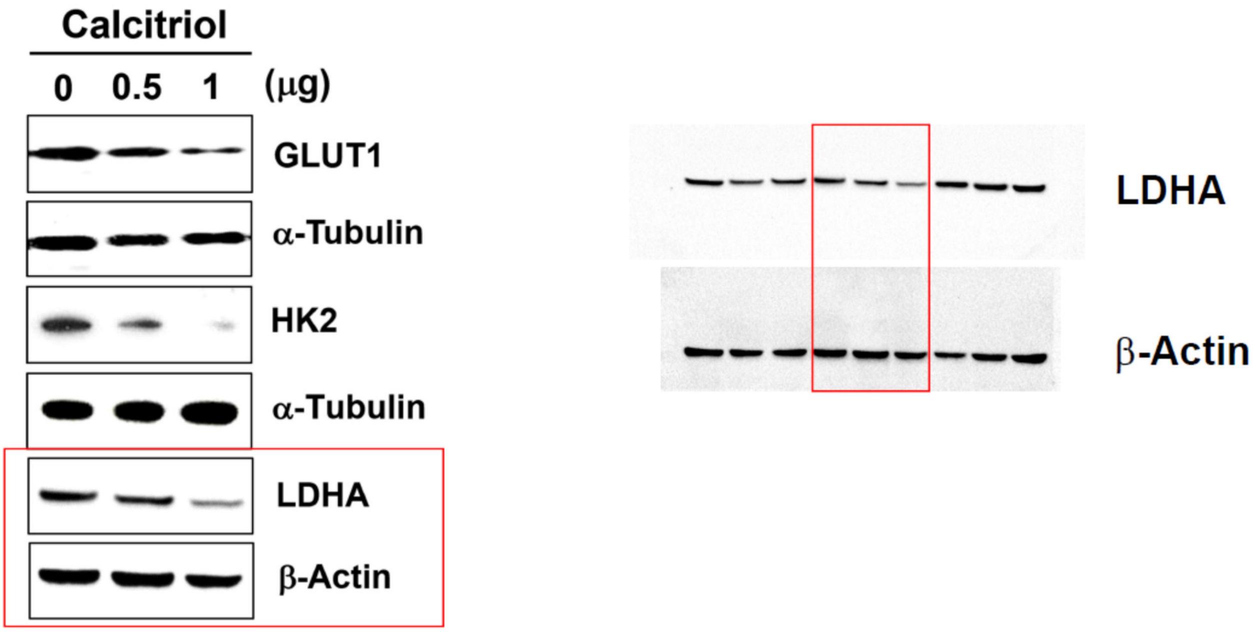

(C)

Figure S3. The original western blotting figures for Figure 3C Western blotting HT29 xenograft: (**A**) GLUT1, α-Tubulin. (**B**) HK2, α-Tubulin. (**C**) LDHA, β-Actin.
